# How do they measure up: Assessing the height of rectal cancer with digital rectal exam, endoscopy, and MRI^,,^^✰^^✰✰^^★^

**DOI:** 10.1016/j.sipas.2022.100096

**Published:** 2022-05-30

**Authors:** Jordan Wlodarczyk, Kshitij Gaur, Nicholas Serniak, Kevin Mertz, Jason Muri, Sarah Koller, Sang W. Lee, Kyle G. Cologne

**Affiliations:** a Division of Colorectal Surgery, Keck School of Medicine, 1510 San Pablo Street, Suite 415, Los Angeles, CA 90033, United States; b Division of Radiology, Keck School of Medicine, Los Angeles, CA, United States

## Abstract

**Background:**

Outcomes in rectal cancer are dependent on tumor height. Modalities for assessing tumor height include MRI, endoscopy, and digital rectal exam (DRE). We seek to identify correlations between these modalities.

**Methods:**

Retrospective analysis of 120 rectal cancer patients at a single institution. Correlation coefficients and distance of the tumor to anal verge between MRI, endoscopy, and DRE were compared by region.

**Results:**

The distances of tumor (cm) from anal verge were: MRI: 6.2 ± 3.0, endoscopy: 5.9 ± 2.9, DRE: 5.4 ± 2.4 (*p* = 0.238). Endoscopy and DRE strongly correlated with MRI (spearman coefficient 0.899 and 0.842, respectively). Endoscopy and DRE also strongly correlated (spearman coefficient 0.876). Correlation coefficients were highest in the middle rectum, weak in the low rectum, and non-correlated in the upper rectum.

**Conclusions:**

MRI, endoscopy, and DRE strongly correlated overall. DRE demonstrated the lowest average distance. Correlations differed by region, suggesting high or low rectal tumors are difficult to characterize.

## Highlights

• MRI, DRE, and endoscopic measurements of rectal tumor height strongly correlate.
• Correlations are strong in the middle rectum, but weak or non-existent otherwise.
• DRE demonstrated the lowest average measurements.
• Low and high rectal tumors may be most difficult to characterize.
• More research is needed to establish a gold standard for assessing tumor height.

## Introduction

The distance of the rectal tumor from the anal verge plays a crucial role in determining treatment course. Oncologic and functional outcomes are dependent, at least in part, on tumor height and the resultant decision to administer neoadjuvant chemoradiotherapy [1,2]. Tumor height also plays a role in determining whether sphincter preservation surgery is possible. For these reasons, the determination of rectal tumor height is of utmost importance in planning both medical and surgical therapy. However, the best way to measure this distance remains controversial.

Despite the importance of determining rectal tumor height, no best practice exists to delineate it. The modalities currently employed generally include flexible sigmoidoscopy, rigid proctoscopy, digital rectal exam, and magnetic resonance imaging (MRI). There is a scarcity of studies examining how well the measurements elicited by these modalities correlate with one another. Studies that do attempt to answer this question have shown that rectal tumor heights vary widely by diagnostic modality, and that there is significant variability between methods [3,4]. Furthermore, the independent accuracies of these methods have yet to be stringently evaluated. While one study suggests that MRI may have the highest inter-observer agreement, and therefore the most potential as a diagnostic tool [5], these claims have yet to be rigorously corroborated. Some studies show that there is still significant variation between radiologist interpretations of MRI height compared to rigid endoscopic evaluation [6].

Because of the paucity of data regarding the best method for determining rectal tumor height, we aimed to compare the measurements of rectal tumor height derived by pelvic MRI (MRI), endoscopy and digital rectal exam (DRE), and to determine whether the general location within the rectum (lower, mid, upper) varied significantly between them. While we believe that the best diagnostic modality will in part be patient-specific, determining inter-modality variability will help define the most accurate method of tumor localization. As these distances may vary by height of the tumor rectum (lower, mid, upper), we also seek to identify any differences when evaluating the different tumor height groups.

## Methods

### Study design

After approval by our IRB, a retrospective review of patients presenting with a diagnosis of rectal cancer to a single institution was undertaken from January 2016 to November 2019. We included patients who had a recorded tumor distance from the anal verge in at least two of the three rectal cancer tumor localization modalities: MRI, DRE, and/or flexible endoscopy. Exclusion criteria included a tumor greater than 15 cm from the anal verge on MRI, and measurement modalities that were post-neoadjuvant therapy.

Flexible endoscopy included either colonoscopy done at an outside hospital or flexible sigmoidoscopy performed by one of four senior board-certified colorectal surgeons at our tertiary referral center, each with a minimum of five years experience after completion of fellowship training. On withdrawal of the scope, tumor height was measured from the lower margin of the tumor to the anal verge. When measurements from both the referring facility's colonoscopy and the operating surgeon's flexible sigmoidoscopy were available, flexible sigmoidoscopy by the operating surgeon was given preference. As rigid proctosigmoidoscopy is inherently different in assessing tumor height than flexible endoscopic tools these patients were excluded from out study.

**Fig. 1:**
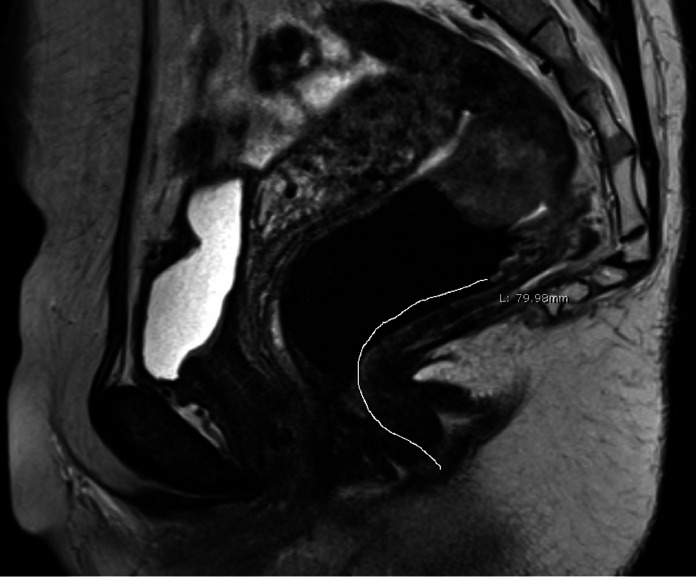
Freehand Interpretation of the Distance of a Rectal Tumor from the Anal Verge.

On MRI, distance from the anal verge to the distal aspect of the lesion was abstracted from the imaging reports from our facility or outside referring center. When this distance was not present in the report, we utilized the tracing tool on Synapse (Fujifilm ® Valhalla, NY) to measure this distance on the mid-sagittal cut of the patient's T2-weighted MRI (Fig. 1). The distal aspect of the tumor was defined by the lowest point of attachment to the rectal wall from where the tumor originated. The anal verge was defined by the transition from hypolucent anoderm to hyperlucent stratified squamous epithelium, which has been demonstrated in the radiologic literature to be a reliable anatomical landmark for the anal verge [7] (Fig. 1). All measurements were taken by a single medical professional who was trained by a senior professor of clinical radiology and medicine to recreate standardized measurement techniques from our institution. Measurements were taken at two separate time points two months apart by the same observer with identical technique. Intraclass correlation coefficient (ICC) was calculated between measurement timepoints to confirm reproducibility of the measurements. An ICC > 0.800 was considered strong correlation between the two measurements

On digital exam, the distance from the distal aspect of the rectal mass to the anal verge was recorded by the physician in their physical exam findings. When physical exam reported DRE measurement from the tumor to the apex of the sphincter complex, the length of the anal canal (as measured on MRI) was added to this number to obtain distance from the anal verge to keep a standard reference point for all modality measurements. If the tumor was not palpable, this patients’ DRE findings were excluded from the study. DRE measurements were made by one of four senior board certified colorectal surgeons at our tertiary referral center, who were the same surgeons performing endoscopy.

If the patient received neoadjuvant chemoradiation, only comparisons between similarly timed assessments were included. For example, pre-neoadjuvant measurement modalities or post-neoadjuvant measurement modalities were compared to each other, but not across neoadjuvant treatment timeframes. This prevented bias from the effect of chemoradiation on tumor shrinkage [5]. Along with tumor height, patients’ age, sex, height, weight, and BMI were abstracted.

All rectal lesions were evaluated as a group, after which they were subdivided into three rectal regions based on prior subdivisions within the literature: lower rectum (<5 cm from the anal verge), middle rectum (5–9.9 cm from the anal verge), and upper rectum (10–15 cm from the anal verge) [8], [9]. These measurements from the anal verge was determined by their distance to the anal verge on MRI.

The primary outcome was defined as the correlation between the different localizing modalities for rectal lesion height in all patient cohorts combined. The secondary outcome was defined as the correlation between the different localizing modalities for rectal lesion height in each defined rectal region.

### Statistical analysis

All data were prepared and compiled using the Statistical Package for the Social Sciences program version 26.0 for Macintosh (SPSS Inc, Chicago, IL, United States). Spearman's correlation coefficient delineated correlation between the different rectal lesion height modalities. A spearman's correlation coefficients (SCC), p-values, and strength of associate was defined as follows: SCC of ≥0.7 and p-value of <0.05- strong correlation; SCC 0.5–0.69 and a p-value of <0.05- moderate correlation; SCC 0.3–0.49 and a p-value of <0.05- weak correlation; SCC <0.3 and a p-value of <0.05- no correlation. An ANOVA with Welch's correction for unequal variance and Bonferroni correction was used to compare means between the three measurement modality groups.

## Results

### Lesions of all rectal locations

120 patients were identified with a diagnosis of rectal cancer with at least two modality measurements. In the entire cohort, the mean age was 60.1 ± 12.3 years, and 63.3% were male. Mean patient height and weight was 165.9 ± 21.9 cm and 77.5 ± 30.3 kg, respectively. The collective median BMI of these rectal cancer patients was 25.4 (interquartile range 8.1). 32.5% (*n* = 39) of patients had two measurement modalities assessing rectal cancer height while the remaining 65.8% (*n* = 79) patients possessed all three modalities. Of the patients with at least two modality measurements, 100% (*n* = 120) of them had an MRI, 34.2% (*n* = 42) of which were images from an outside facility. Of these 42 patients with outside hospital MRI only 38.1% (*n* = 16) had written reports containing the height of the tumor. 91.7% (*n* = 110) of the total cohort had an endoscopic report reporting distance from the anal verge (of which 85.8%, *n* = 94, were form our institution), and 72.5% (*n* = 87) had physical exam findings recorded in their note containing a DRE detailing measurement from the tumor to the anal verge or pelvic floor. 16.7% (*n* = 20) of patients had a DRE recorded in the medical records but were unable to palpate the tumor. 80% (*n* = 16) of these unpalpable tumors on DRE were located 10–15 cm from the anal verge on alternative measurement modalities. 21.8% (*n* = 19) of these DRE tumor height measurements were to the pelvic floor for which their anal canal was measured for determination of tumor height to the anal verge as outlined above for standardization. (Table 1) Patients without palpable tumor on DRE were excluded from analysis.

**Table 1:** Patient Demographics.

	*Lower rectum (< 5* *cm)*	*Middle rectum (5 to 9.9* *cm):*	*Upper rectum (10 to 15* *cm):*	*Total Cohort*
Number	36	54	30	120
Age (years)	61.1 ± 13.1	59.0 ± 11.7	60.7 ± 12.6	60.1 ± 12.3
BMI	25.7 (IQR: 6.5)	25.9 (IQR: 8.0)	23.7 (IQR: 11.7)	25.4 (IQR: 8.1)
Percent Male	61.1%	70.4%	53.3%	63.3%
Height (cm)	169.4 ± 10.7	165.5 ± 23.0	162.6 ± 28.	165.9 ± 21.9
Weight (kg)	74.8 ± 21.8	79.7 ± 37.8	76.7 ± 23.5	77.5 ± 30.3
Height on MRI (cm)	3.2 ± 1.1	7.2 ± 1.4*^,^$	12.1 ± 2.0	6.2 ± 3.0
Height on Endoscopy (cm)	3.6 ± 1.4	6.6 ± 2.0$	11.0 ± 2.4	5.9 ± 2.9
Height on DRE (cm)	3.4 ± 1.3	6.1 ± 1.3*	9.5 ± 2.5	5.4 ± 2.4

⁎ Statistically different (*p* = 0.035).

$ Statistically different (*p* = 0.049).

**Fig. 2:**
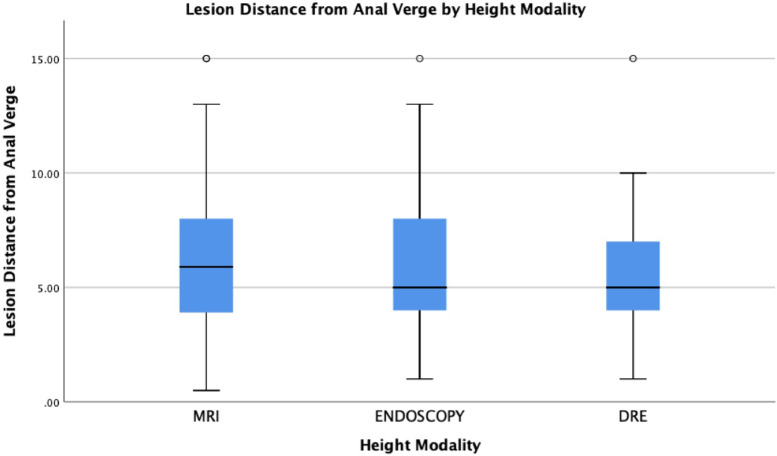
Average Rectal Tumor Distance from the Anal Verge by Measurement Modality (cm).

The average distance of the tumor from the anal verge, measured in 79 individuals who had all three measurement modalities, were MRI: 6.2 ± 3.0 cm, endoscopy: 5.9 ± 2.9 cm, and DRE: 5.4 ± 2.4 cm, respectively. The difference between the distance measurements for all three modalities was not statistically significant (*p* = 0.238) (Fig. 2). The ICC between measurements make on MRI was 0.893 which demonstrated strong correlation.

**Fig. 3:**
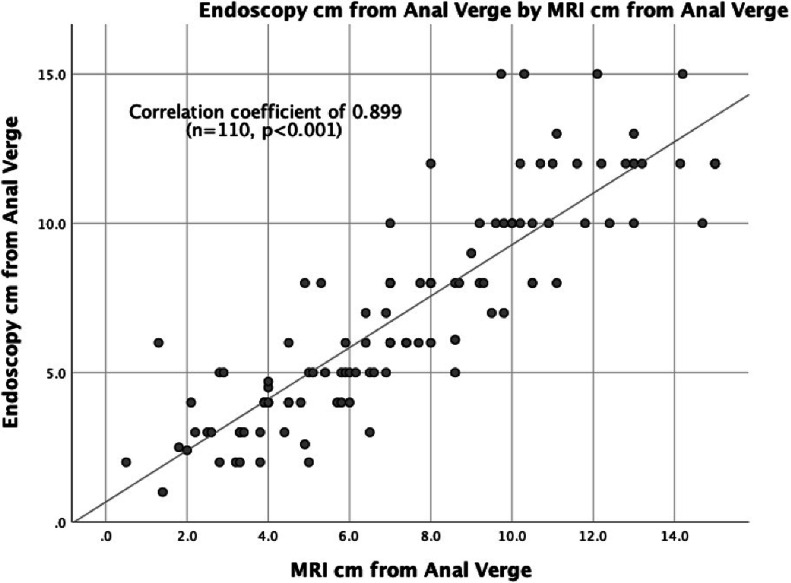
Tumor Height Correlation: MRI and Endoscopy (cm).

**Fig. 4:**
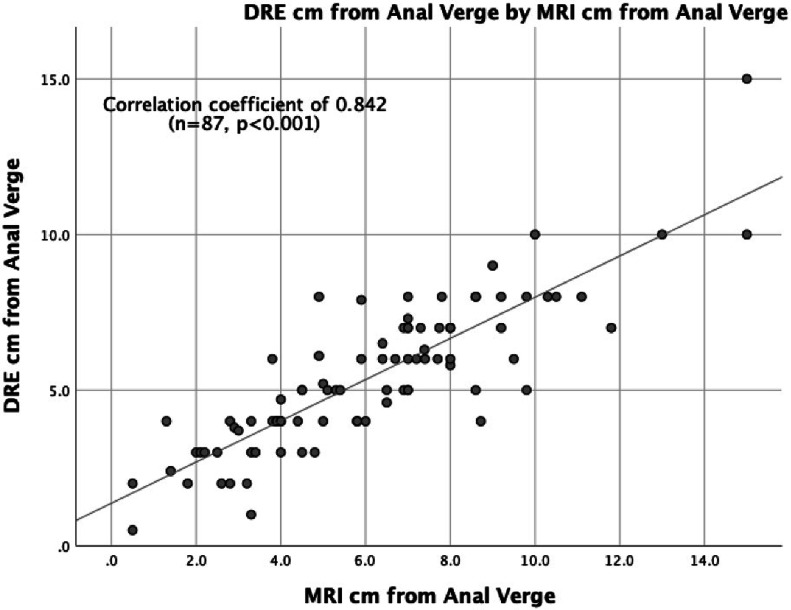
Tumor Height Correlation: MRI and DRE (cm).

**Fig. 5:**
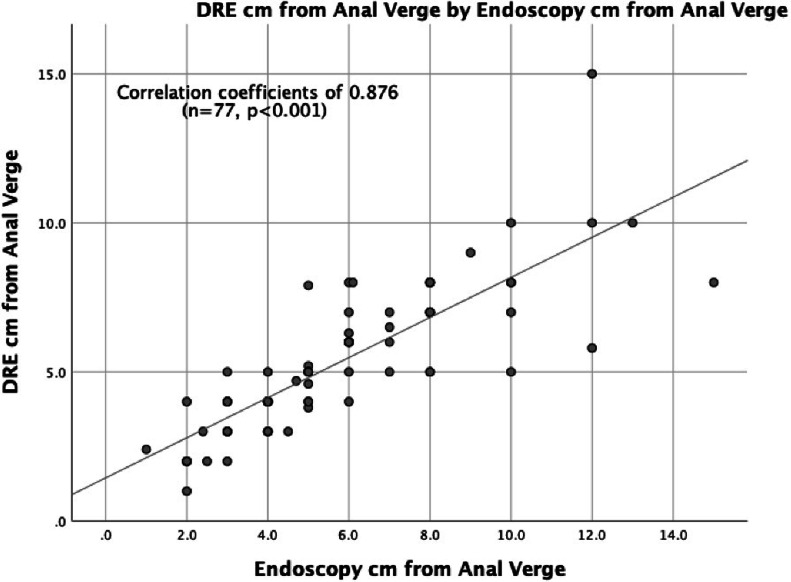
Tumor Height Correlation: Endoscopy and DRE (cm).

For all rectal cancer cases, when compared to MRI, endoscopy and DRE were strongly correlated with tumor height, with SCCs of 0.899 (*n* = 110, *p* ≤ 0.001) and 0.842 (*n* = 87, *p* ≤ 0.001), respectively (Fig. 3, Fig. 4). When endoscopy was directly compared to DRE, the measurements again strongly correlated with tumor height, with a SCC of 0.876 (*n* = 77, *p* ≤ 0.001) (Fig. 5).

### Lesions of the lower rectum (< 5 cm)

Thirty-six patients were identified with a lesion height of < 5 cm. The average age of these patients was 61.1 ± 13.1 years, and 61.1% were male. Their mean height and weight was 169.4 ± 10.7 cm and 74.8 ± 21.8 kg, respectively. The collective median BMI of these rectal cancer patients was 25.7 (interquartile range 6.5).

The average distance of the tumor from the anal verge, measured in 30 individuals who had all three measurement modalities, were MRI: 3.2 ± 1.1 cm, endoscopy: 3.6 ± 1.4 cm, and DRE: 3.4 ± 1.3 cm, respectively. The difference between the distance measurements for all three modalities was not statistically significant (*p* = 0.889).

When compared to MRI, endoscopy was weakly correlated with tumor height, with a SCC of 0.416 (*n* = 32, *p* = 0.018), and was moderately correlated with DRE with a SCC 0.643 (*n* = 34, *p* ≤ 0.001). When endoscopy was directly compared to DRE the measurements strongly correlated with tumor height, with a SCC of 0.754 (*n* = 30, *p* ≤ 0.001).

### Lesions of the middle rectum (5 to 9.9 cm)

Fifty-four patients were identified with a lesion height of 5.0 to 9.9 cm. The average age of these patients was 59.0 ± 11.7 years, and 70.4% were male. Their mean height and weight was 165.5 ± 23.0 cm and 79.7 ± 37.8 kg, respectively. The collective median BMI of these rectal cancer patients was 25.9 (interquartile range 8.0).

The average distance of the tumor from the anal verge, measured in 39 individuals who had all three measurement modalities, were MRI: 7.2 ± 1.4 cm, endoscopy: 6.6 ± 2.0 cm, and DRE: 6.1 ± 1.3 cm, respectively. The difference between the distance measurements for all three modalities on ANOVA demonstrated statistically significance (*p* = 0.018). When individual comparisons were evaluated, the difference in measurements between MRI and DRE and MRI and endoscopy were statistically significant (*p* = 0.035 and *p* = 0.049, respectively), while the difference in measurement DRE and endoscopy (*p* = 1.00) was not.

When compared to MRI, endoscopy strongly correlated with tumor height with a SCC of 0.719 (*n* = 48, *p* ≤ 0.001) and weakly correlated with DRE, with a SCC of 0.481 (*n* = 45, *p* = 0.001). When endoscopy was compared to DRE, the measurements moderately correlated with tumor height, with a SCC of 0.585 (*n* = 39, *p* ≤ 0.001).

### Lesions of the upper rectum (10 to 15 cm)

Thirty patients were identified with a lesion height of 10 to 15 cm. The average age of these patients was 60.7 ± 12.6 years, 53.3% were male. Their mean height and weight was 162.6 ± 28.8 cm and 76.7 ± 23.5 kg, respectively. The collective median BMI of these rectal cancer patients was 23.7 (interquartile range 11.7).

The average distance of the tumor from the anal verge, measured in 10 individuals who had all three measurement modalities, were MRI: 12.1 ± 2.0 cm, endoscopy: 11.0 ± 2.4 cm, and DRE: 9.5 ± 2.5 cm, respectively. The difference between the distance measurements for all three modalities was not statistically significant (*p* = 0.705).

When compared to MRI, endoscopy did not correlate with tumor height, with a SCC of 0.353 (*n* = 30, *p* = 0.056) and also did not correlate with DRE, with a SCC of 0.428 (*n* = 8, *p* = 0.290). When endoscopy was compared to DRE, the measurements moderately correlated with tumor height, with a SCC of 0.370 (*n* = 8, *p* = 0.368).

## Discussion and conclusion

Despite advances in clinical tools used to stage rectal tumors, very little data exists comparing the precision and accuracy between measurement modalities, particularly when stratified by low, middle, or upper rectum. The studies that do exist comparing correlation between modalities when stratifying by region do not comprehensively compare correlations between all 3 modalities to inform which tools have the highest utility by region [10]. Our study, evaluated heights obtained from MRI, endoscopy, and DRE and analyzed correlations between all 3 tools at each rectal region to improve our understanding of which modality may be most precise when stratified by region. We demonstrated strong correlation between all tumor height assessment modalities when all rectal cancer patients were grouped together. DRE overall demonstrated the lowest average distance measurement, perhaps representing an underestimation of the height of rectal tumors, though statistical significance was only established in the middle rectal tumor group. We anticipate that if our upper rectal cancer group had more power similar results would have been demonstrated.

When evaluating the tumor height assessment modalities across rectal lesion groups, MRI and endoscopy had the strongest correlation in the middle rectal tumor group. This correlation was weak in the lower rectum and non-existent in the upper rectum. This finding suggests that the best rectal region for measuring tumor height by endoscopy may be in the middle rectum. This strong correlation has not been consistently demonstrated in the previous literature. MRI and endoscopy have previously been found to disagree with one another. Previous studies have been discordant in their findings demonstrating both overestimation and underestimation of MRI tumor height as compared to endoscopy across different studies [3,4]. Only very few studies have demonstrated that MRI tumor height measurements correlated well with rigid proctosigmoidoscopy [11]. In our study MRI, while possessing a strong correlation, varied in the middle rectal region with demonstrated overestimation of tumor height as compared to endoscopy. MRI measurement of rectal tumors typically measures along the wall of the rectum (a curved surface), whereas endoscopy and DRE measurement is performed in a straight line, which may account for this apparent overestimation. Furthermore, the use of insufflation, compression of the anal canal and other endoscopist related variability may account for these differences and suggest that endoscopy may be best used for middle and upper rectal cancers. Our own practice is to take a picture of the tumor with the scope partially within the anal canal as well as (if possible) a retroflexion view. This helps visualize relationships to the apex of the sphincter, which aids in operative planning but is very difficult to compare measurements on.

When MRI and DRE were compared, the strongest correlation was present in the low rectal tumors group. As tumor height increased, the correlation decreased and eventually disappeared with middle rectal tumors and high rectal tumors, respectively. Because neither endoscopy nor DRE correlated well with MRI for high rectal tumors, clinical utility of endoscopy and DRE in delineating tumor height appears to be limited in this region.

Finally, endoscopy and DRE demonstrated the strongest correlation in the low rectal tumors group. The correlation between endoscopy and DRE decreased and eventually disappeared as tumor height increased, similar to the correlation between MRI and DRE. This finding is not surprising, as the length limitations of a digit may hinder its ability to reach greater heights. Current literature supports the increased accuracy of DRE as well as concordance with endoscopy with lower tumor location [12,19]. Tumor characteristics, such as fixation to surrounding structures, may change the height of the tumor further contribute to discrepancy in measurements.

From a clinical relevance standpoint, small differences in measured tumor height rarely affect clinical decision making unless present in very low or high rectal cancers. For example, in mid rectal tumors if two different imaging modalities measure a tumor at 7.2 cm versus 6.1 cm this will not influence the decision for neoadjuvant therapy or surgical approach. For high rectal tumors, it is key for these measurement modalities to distinguish between rectal, rectosigmoid and sigmoid tumors as this impacts the ability to treat with neoadjuvant therapy. As these lesions are typically over 12–15 cm from the anal verge these lesions are not palpable on digital rectal exam. We observed this in our study as well as most patients with an MRI located lesion over 12 cm had no mention of distance or we noted to be not palpable on DRE. Additionally in the very lowest rectal tumors the ability to preserve the sphincters at time of surgery is key and may in fact depend on differences of half a centimeter. The ability for our study to compare all these measurements gives the reader a helpful understanding of what effect small differences in these measurement modalities confer.

Our study suffers from several limitations. Our small sample size is underpowered to adequately determine correlation of measurement modalities within the different sub-groups of the upper, middle, and lower rectum. Furthermore, selecting MRI as the reference measurement for DRE and endoscopy is prone to some subjectivity. Even within guidelines created by radiologic societies, identification of the anal verge on MRI is a controversial topic. It is not clear how accurate the radiologic localization of the anal verge correlates to the physical localization of the anal verge on digital or endoscopic assessment. Further, other studies have utilized multiple straight lines vs. a singular straight line vs. a singular curvilinear line on sagittal MRI to measure rectal tumor height, with no clear consensus as to the superior approach [13], [14], [15], [16], [17], [18]. Our study utilized a singular curvilinear line, which demonstrates acceptable accuracy but is more difficult to recreate between observers [7].

Endoscopy has its own limitations when assessing tumor height. The classical teaching is that rigid endoscopy is more accurate than flexible endoscopy for assessing tumor height. In a study comparing rigid endoscopy and flexible endoscopy to DRE, the two modalities demonstrated acceptable agreement in identifying the height of rectal cancer overall, but the discrepancy between rigid and flexible endoscopy exceeded two centimeters when the lesion was above five centimeters from the anal verge [19]. Our group's preference was to use flexible endoscopy due to its superior visualization of the rectum in general. Differences in rectal height measurements could also vary depending on the measuring endoscopist. The instillation of intra-rectal gas could distend the rectal wall artificially extending the tumor height. The endoscopists usually measure the distance with the rectum inflated, which may lead to overestimate the tumor height by raising both the tumor and the peritoneal refection.

It is likely there is no perfect measurement modality to assess the height of rectal cancer, thus underscoring the importance of multidisciplinary tumor board discussions regarding the timing and type of neoadjuvant chemotherapy and radiation. Other factors that may influence that decision but that are not addressed in this study include distance to the mesorectal envelope and high-risk characteristics such as involvement of extra-mesorectal lymph nodes or extra-mural venous invasion. The delineation of tumor location is an important component of medical and surgical decision-making and relationships between height assessment modalities should be completely understood.

As a whole, there is strong correlation between MRI, endoscopy and DRE when measuring the distance of rectal tumors from the anal verge. When separating the rectum into different zones, MRI and endoscopy are most correlated in the middle rectum, while MRI and DRE are most correlated in the lower rectum. Further study identifying the accuracy of the three modalities is needed to identify the most accurate method for tumor height assessment as there is no clear gold standard.

### Availability of data and materials

Data available upon request.

### Code availability

Not applicable.

### Ethics approval

IRB approval was granted for the completion of this study with the ID number HS-17–00,058

### Consent to participate

Not applicable.

### Consent for publication

All authors listed on this manuscript have consented for publication of this manuscript in International Journal of Colorectal Disease.

## Declaration of Competing Interest

The authors declare that they have no known competing financial interests or personal relationships that could have appeared to influence the work reported in this paper.

## Appendix Supplementary materials

Image, application 1
