# Supplementary material for: How do they measure up: Assessing the height of rectal cancer with digital rectal exam, endoscopy, and MRI,,
Source: Surg Pract Sci. 2022 May 30;10:100096. doi: 10.1016/j.sipas.2022.100096 (PMC11749183; doi:10.1016/j.sipas.2022.100096)
Supplement: Supplementary file 1 [file mmc1.docx]

BACKGROUND:

Outcomes in rectal cancer are dependent on tumor height. Modalities for assessing tumor height include MRI, endoscopy, and digital rectal exam (DRE). We seek to identify correlations between these modalities.

METHODS:

Retrospective analysis of 120 rectal cancer patients at a single institution. Correlation coefficients and distance of the tumor to anal verge between MRI, endoscopy, and DRE were compared by region.

RESULTS:

The distances of tumor (cm) from anal verge were: MRI: 6.2 ± 3.0, endoscopy: 5.9 ± 2.9, DRE: 5.4 ± 2.4 (p=0.238). Endoscopy and DRE strongly correlated with MRI (spearman coefficient 0.899 and 0.842, respectively). Endoscopy and DRE also strongly correlated (spearman coefficient 0.876). Correlation coefficients were highest in the middle rectum, weak in the low rectum, and non-correlated in the upper rectum.

CONCLUSIONS:

MRI, endoscopy, and DRE strongly correlated overall. DRE demonstrated the lowest average distance. Correlations differed by region, suggesting high or low rectal tumors are difficult to characterize.
